# Role of Socioeconomic Indicators on Development of Obesity from a Life Course Perspective

**DOI:** 10.1155/2009/625168

**Published:** 2009-06-07

**Authors:** Minna K. Salonen, Eero Kajantie, Clive Osmond, Tom Forsén, Hilkka Ylihärsilä, Maria Paile-Hyvärinen, David J. P. Barker, Johan G. Eriksson

**Affiliations:** ^1^Department of Health Promotion and Chronic Disease Prevention, National Institute for Health and Welfare, 00300 Helsinki, Finland; ^2^MRC Epidemiology Resource Centre, University of Southampton, Southampton SO16 6YD, UK; ^3^Heart Research Center, Oregon Health and Science University, Portland, OR 97239, USA; ^4^Department of General Practice and Primary Health Care, University of Helsinki, P. O. Box 20, 00014 Helsinki, Finland; ^5^Unit of General Practice, Helsinki University Central Hospital, P. O. Box 705, 00029 HUS, Finland

## Abstract

*Aims*. Development of obesity is modified by several factors, including socioeconomic ones. We studied the importance of socioeconomic indicators on the development of obesity from a life course perspective. *Methods*. 2003 people born 1934–1944 in Helsinki, Finland, participated in clinical examinations in 2001–2004. Obesity was defined as body mass index (BMI) >30 kg/m^2^. *Results*. Prevalence of obesity was 22.3% in men and 27.2% in women. Lower educational attainment and lower adult social class were associated with higher BMI in both men (*P* = .03 and *P* < .01) and women (*P* < .001 and *P* = .01). Childhood social class was inversely associated with BMI only in men (*P* < .001); lower household income was associated with higher BMI in women only (*P* < .001). Those men belonging to the lowest childhood social class had higher risk of being obese than those of the highest childhood social class (OR 1.8 (95% CI: 1.0–3.1)). Household income was the strongest predictor of obesity among women. *Conclusion*. Overweight and obesity are inversely associated with socioeconomic status. Men seem to be more susceptible to adverse childhood socioeconomic circumstances than women, while adult socioeconomic indicators were more strongly associated with obesity in women.

## 1. Background

Obesity is one of the most important risk factors contributing to the overall disease burden worldwide [1]. The major causes of premature morbidity and mortality and public health concerns associated with obesity are cardiovascular disease, type 2 diabetes, and certain cancers [2]. The risk of, for example, type 2 diabetes begins to increase already from a BMI of ~21.0 kg/m^2^, thereby reducing life expectancy and greatly increasing the health and societal burdens [3].

Obesity is known to track from childhood into adult life [4, 5], and children who have a higher body mass index are at increased risk of becoming obese in adult life. Although genetic and lifestyle factors are important, socioeconomic circumstances are closely linked to adult overweight and obesity [6–8]. Not only socioeconomic circumstances per se, but also a continuous widening of these differences as reported in several studies induce serious concerns in the field of public health [9–11].

The concept of socioeconomic status (SES) and socioeconomic position (SEP) refers to the social and economic factors influencing the position individuals or groups hold within the societal structure [12, 13]. There are several indicators of socioeconomic status; the most commonly used are occupation, education and income [12–14]. One way to increase our knowledge of the importance of socioeconomic circumstances in obesity is by using several indicators of socioeconomic status, including early life experiences as well as more proximal ones of socioeconomic status [15–17].

From a life course perspective parental occupation can be used as an indicator of childhood socioeconomic status, whereas an individual's own occupation in adulthood can be seen as a reflection of his/her own societal position. Education can be seen as a useful indicator of socioeconomic status from early adult life on, since formal education is usually completed in young adulthood [18, 19]. Furthermore level of education is considered to remain rather stable through the life course. Income again is the most commonly used indicator of socioeconomic status measuring economic resources. Income can be used as a measure of the socioeconomic status through adulthood although time-related variability of income should be taken into account especially when using longitudinal study designs but also when other socioeconomic indicators are not available.

Income mainly affects health through a direct effect on resources available that are alternately mediated by more indirect factors in the causal chain such as life style and behaviours [20].

However, poor health may also affect income. It is suggested that excess weight may have detrimental effects on employment opportunities and that among people with same level of education obese, people tend to have significantly lower income than normal weight people [21, 22]. Marital status is considered as an environmental or sociodemographic rather than socioeconomic predictor. It has been observed that being married is predictor of higher weight gain primarily in women [23].

A strong consistent relationship has been observed between low socioeconomic status in early life and overweight and obesity in adulthood in developed countries [7, 8]. Most of the studies focusing on socioeconomic status and obesity have been cross-sectional or they have had a limited period of follow-up. Therefore a true life course impact of indicators of socioeconomic status on obesity is less well known [7, 8].

## 2. Aim of the Study

The aim of this research project was to study how different indicators of socioeconomic status, including parental occupation (used as a marker of childhood SES), participants' educational attainment, occupation, household income, and marital status are related to obesity among Finnish men and women in later life using a longitudinal study design.

## 3. Methods

This study is part of the Helsinki Birth Cohort Study which includes 8760 individuals, 4630 men and 4130 women, born in Helsinki during the years 1934 to 1944, who attended child welfare clinics in the city of Helsinki and who were still resident in Finland in 1971. Of the original study cohort, 77% also went to school in Helsinki. Details of the birth, child welfare, and school health records have been described in detail before [24].

A unique personal identifier has been assigned to every resident of Finland by the year 1971. Using this identification number we traced people belonging to the cohort. All 7079 people belonging to the original epidemiological cohort, who were still alive and resident in Finland, were sent a questionnaire in the year 2000. A total of 4515 individuals provided adequate data and allowed the research group to contact them.

A subset of 2902 men (*n* = 1277) and women (*n* = 1625) were contacted and invited to participate in a clinical examination during the years 2001–2004. They were selected from the initial study population using random number tables as previously described [25]. Of them 2003 men (*n* = 928) and women (*n* = 1075) participated at an average age of 61.5 years.

The clinical study protocol was approved by the Ethics Committee of Epidemiology and Public Health at the Hospital District of Helsinki and Uusimaa. Written informed consent was obtained from each subject.

Height was measured to the nearest 0.1 cm, and weight was measured to the nearest 0.1 kg. Body mass index (BMI) was calculated using information on body height and weight (weight [kg]/height [m^2^]) from the clinical examination. Subjects were considered obese when their body mass index was ≥30 kg/m^2^.

Using father's occupation, which was available from the birth and child welfare clinic and school records, we grouped the fathers into three groups (labourers, lower middle class, and upper middle class) originally based upon a social classification system used by Statistics Finland [26]. The main levels of social classification from the top are upper-level employees with administrative, managerial, professional and related occupations, lower-level employees with administrative and clerical occupations, manual workers, pensioners, students, and lastly others (unemployed and persons of unknown occupation). We defined upper-level employees as upper middle class. Middle class consists of lower-level employees, and the labourers' class includes the manual workers.

Childhood social class was based on the father's highest social class which was based upon his occupation. Overall 59.6% of the fathers were labourers, and 26.5% were classified as lower middle class. 13.3% of the fathers belonged to the upper middle class. Pensioners, students, unemployed subjects, and persons with unknown occupation were excluded from the analysis because they could not be classified in a homogenous occupational category. Childhood socioeconomic status of 0.7% of the cohort was unclassified or missing.

Through Statistics Finland we obtained data on the study subjects' own occupation as recorded in 1990 Census and they were in a similar way regrouped into three categories. Upper middle class includes upper-level employees (*n* = 607). Middle class consists of lower-level employees (*n* = 978), and the labourers' class includes the manual workers (*n* = 299). 119 subjects from the cohort were unclassified, 59 of these were retired, and the rest were unemployed and people of unknown socioeconomic status. The socioeconomic distribution within the city of Helsinki in the year 1990 was similar to that of our cohort although there is a slight overrepresentation of the higher social class in our study material [27].

Through questionnaire, which was a part of the clinical examination, we obtained data on schooling (number of years in school). Educational attainment was categorized into three groups according to number of years in schools: basic (≤9 years of education); secondary (10–12 years); and higher (≥13 years of education). Information on marital status was also obtained from the questionnaire. Data on taxable household income in Finnish marks per year in the year of 1990 were obtained from the Statistics Finland. The basic data are drawn from the Tax Administration's database and are based on total data including all persons who have received income subject to taxation or own property subject to taxation.

Socioeconomic and sociodemographic predictors were parental and own social class based on occupation, educational attainment, household income, and marital status. Tests for trends in body mass index were based on linear regression models, always including age. We also calculated the odds ratios for obesity in relation to estimates of childhood and adult social class, educational attainment, and household income. After fitting age-adjusted base models including individual effects of each socioeconomic indicator, we added the indicator of childhood socioeconomic status (childhood social class), then socioeconomic indicators in adulthood (educational attainment, adult social class, household income), and lastly marital status. Also the correlations between the indicators were studied. Because previous studies have shown more consistent socioeconomic patterning of obesity in women than in men; the analyses were conducted for men and women separately. Tests for trends in body mass index were based on the corresponding linear regression models. Statistical analyses were made by SPSS 13.0 software.

## 4. Results

Mean BMI was 27.5 kg/m^2^ in men and 27.7 kg/m^2^ in women. Of the men 73.8% and 67.6% of the women were overweight or obese. Men were more often overweight while women were more commonly obese. The prevalence of obesity (BMI ≥ 30 kg/m^2^) was 22.3% in men and 27.2% in women. Table 1 shows prevalence of overweight and obesity in the study group.

BMI was highest in people who belonged to the lowest social class in childhood as well as in adult life and who had only a basic level of education. Table 2 shows mean BMI according to social class (in childhood and in adulthood), educational attainment, household income, and marital status. In men social class both in childhood and adulthood as well as educational attainment were all inversely associated with BMI. Among men there was no association with household income. In women the results were similar to those of the men except for childhood social class that did not reach statistical significance (*P* = .10). Household income showed a strong inverse association with BMI in women.

In men, when the socioeconomic indicators were examined one by one adjusted for age, all but household income was associated with later obesity (Table 3). We used multiple logistic regression models to study the independent effects of the indicators, since correlations between the socioeconomic indicators were only modest (range 0.13–0.50). Introducing the indicators of adult socioeconomic status (educational attainment and adult social class) into the model slightly weakened the association between childhood social socioeconomic status and obesity. Educational attainment and adult social class were no longer associated with obesity. In the final model including all the indicators mentioned above and additionally household income, childhood socioeconomic status was the only indicator that remained associated with obesity among men.

There was no linear association between BMI and childhood social class among women. However, Table 4 shows that those belonging to the middle or lower childhood social class had slightly greater risk of being obese compared to those of the higher class with OR 1.7 (95% CI: 1.0–2.8) and OR 1.6 (95% CI: 1.0–2.6), respectively. The indicators of adult socioeconomic status were all associated with obesity. When educational attainment was included into the model, childhood social class was no longer associated with obesity. Furthermore after introducing adult social class into the model, most of these associations were attenuated, and finally household income was the only indicator remaining significantly associated with obesity in women.

## 5. Discussion

The prevalence of overweight and obesity in the study sample was high, being 73.9% among males and 67.7% among females. This high prevalence is consistent with previous findings [2, 10, 28]. When examining childhood and adult socioeconomic indicators separately, all indicators of low socioeconomic status (i.e., childhood and adult social class, educational attainment, and income) were associated with obesity in both genders. Household income, however, was associated with obesity only in women. There was no association between marital status and obesity. After further adjustments childhood socioeconomic status seemed to be of greater importance among men, while adult socioeconomic indicators more strongly predicted obesity in women. The present findings of the associations between adverse socioeconomic circumstances and poor health outcomes are consistent with the previous studies in Finland, Europe, and North America [29].

Our aim was to study how socioeconomic indicators during the life course are associated with obesity. A traditional life course approach model takes into account the possibility of critical periods in early life but emphasises the accumulation of risk factors resulting from exposure to adverse environments during childhood, adolescence, and adulthood [30]. We have based this work on previous ones using a similar approach [15–17]. The particular socioeconomic approach in this study is the use of several indicators of socioeconomic status from childhood to adulthood. Such an approach has advantages and helps improving our understanding of the socioeconomic background of obesity.

The inverse relationship between childhood socioeconomic status and obesity in adult life has been previously reported [8, 16, 17]. Childhood socioeconomic status may also influence adult health independently of adult socioeconomic status [29, 30]. In our study the association between childhood socioeconomic status and obesity in men remained highly significant, and only slightly weakened after adjusting for adult socioeconomic indicators. None of the adulthood socioeconomic indicators was associated with obesity in multivariate analysis among men. A similar tendency for childhood social class was found in women. In women the association between childhood social class and obesity disappeared after adult socioeconomic indicators were included into the model. In the final model, including both childhood and adult socioeconomic indicators, only income remained statistically significantly associated with obesity in women.

Health related knowledge and skills can be seen as a reflection of educational attainment and consequently education might have a major impact in predicting health-related behaviours such as dietary habits and physical activity [31, 32]. In a multiple regression model—including both childhood social class and education—education remained statistically significant among women, while childhood social class was associated with obesity in men.

Occupation is commonly used as a measure of socioeconomic status in Europe while income or education is more frequently used in the United States [33]. Education and income should not be considered interchangeable. Standard measures of education and income are correlated; these correlations appears to be too weak to justify using education as a proxy for income or vice versa. In our study correlation between education and income was modest (*r*_*s*_ = 0.26). On the other hand, occupational categories based on prestige, skills, social influence, and power have been the primary bases for socioeconomic classification in Western Europe. Even though studies have frequently shown a strong relationships between occupational status and diverse health indicators [33], also after controlling income or education [34], some critique have emerged, for example, because of difficulties on constructing adequate hierarchical classifications [33] again supporting the use of multiple socioeconomic indicators in epidemiological research.

In the present study paternal occupation was used to assess childhood socioeconomic status. Quality of the information based on the records was somewhat diverse and occasionally caused difficulties to attain the adequate classification. However, the same problems have been confronted previously [35, 36]. In addition distribution of the childhood socioeconomic status was skewed. The lowest class comprising 60% of the fathers is close to the known prevalence of labourer families in and around Helsinki being ~60% at that time. Thus, the socioeconomic status distribution of our sample may be similar to that of the city as whole. Even though the societal construction has changed in many aspects during the past decades and, in our case, affected content of occupations, for consistency we chose to use equal classification concerning adult and parental occupation when comparing these two.

Income is one of the best indicators to measure material resources, although commonly because of the lack of this information, other indicators have been used as a substitute [12]. While higher educational attainment through increased knowledge enables an individual to make healthy choices regarding diet and physical activity, economic constraints may restrict behavioural options in this aspect [32]. We used household income, as a proxy for material resources, based on the information from the Finnish taxation register. We chose to use the taxation year 1990 since presumably in this study population, aged 46–56 at the time, income during this period of life was sustainable and steady. Only 20 people (1%) experienced unemployment during the year in question.

Besides the interrelationships between different socioeconomic indicators there may be complex interactions between socioeconomic status and obesity; socioeconomic status may influence obesity; obesity may influence socioeconomic status, respectively. Furthermore there may be critical periods during childhood making children more vulnerable to the adverse physical, psychological, and social environments.

The longitudinal study setting is certainly a major strength of our study, however there are some limitations that need to be addressed. We have studied people who were born in the Helsinki University Central Hospital. The people in our study may, therefore, not be representative of all people born in Helsinki. However at that time in Helsinki ~60% of men worked as labourers, which is similar to the percentage of fathers in our study who belonged to this social class. Another important fact to keep in mind is that it is not known how applicable these findings are to children of today. However, we feel that the findings add information on the importance of socioeconomic factors in the development of obesity among obese adults of today. Furthermore we did not have information on the SES of the community of the participants, which would have added strength to the paper. It is of particular note that the people in our study were born or growing up during the period around Second World War. This time period may have created its own challenges on health in different ways regardless of their socioeconomic circumstances. For example, food restrictions may have effected growth of the children. The present study does not have adequate power to study the specific effects of different periods of war on adult obesity. However, on the whole, the impact of the Second World War on growth in this cohort was not large [37].

## 6. Conclusions

Along with these results we conclude that the key indicators of socioeconomic status such as parental occupation and own occupation, educational attainment, and household income are associated with adult obesity. We also conclude that higher socioeconomic status achieved in later life does not fully compensate the nonoptimal childhood circumstances on obesity in later life. These findings also suggest that men may be more vulnerable to the adverse childhood circumstances than women. Income was not associated with obesity among men. In women income was and remained statistically significantly associated with obesity also after further adjustments.

Finally we conclude that obesity at age 60 to 70 years is associated with low socioeconomic status in both childhood and adulthood. In men, the effects of childhood socioeconomic status are independent of socioeconomic status in adulthood, while in women these effects are attenuated when adjusted for adult socioeconomic status, in particular as indicated by income.

These findings stress the importance of a life course concept when focusing upon risk factors for adult noncommunicable diseases.

## Figures and Tables

**Table 1:** Prevalence of overweight and obesity according to BMI at age 57–70 years among 927 men and 1074 women.

		*Men*	*Women*
	BMI(kg/m^2^)	%	%
Underweight	<18.5	—	0.6
Normal	18.5–24.9	26.1	31.7
Overweight	25.0–29.9	51.5	40.4
Obesity, class I	30.0–34.9	17.3	18.4
Obesity, class II	35.0–39.9	3.8	6.5
Obesity, class III	≤40	1.2	2.3

**Table 2:** Mean body mass index (BMI) and 95% confidence intervals (95% CI) according to socioeconomic status in childhood and adulthood among men and women aged 61.5 (SD ± 2.9) years. *P*-value for linear trend (adjusted for age).

	*Men*			*Women*		
	*n* = 920			*n* = 1067		

*Social class in childhood*	*BMI*	*95*% *CI*	*n*	*BMI*	*95*% *CI*	*n*
Lower	28.0	(27.6–28.3)	525	27.9	(27.5–28.3)	667
Middle	27.3	(26.8–27.8)	254	27.7	(27.1–28.3)	276
Higher	26.5	(25.9–27.1)	141	26.9	(26.2–27.7)	124
*P* * for trend*	<.*001*			*.10*		
*Education*						
Basic	28.1	(27.6–28.6)	256	28.3	(27.7–28.8)	299
Secondary	27.3	(26.8–27.8)	262	28.1	(27.6–28.6)	347
Higher	27.3	(26.9–27.8)	388	26.9	(26.4–27.4)	402
*P* * for trend*	*.03*			<.*001*		
*Social class in adulthood*						
Lower	28.4	(27.6–29.1)	191	28.9	(27.9–29.9)	108
Middle	27.5	(27.1–27.9)	333	27.8	(27.4–28.2)	643
Higher	27.0	(26.6–27.4)	350	26.8	(26.2–27.4)	257
*P* * for trend*	<*.01*			*.01*		
*Household income*						
Lowest quartile	27.7	(27.0–28.3)	166	28.1	(27.5–28.7)	332
2nd quartile	27.7	(27.2–28.3)	229	28.4	(27.8–29.0)	262
3rd quartile	27.4	(27.0–27.8)	272	27.5	(26.8–28.2)	233
Highest quartile	27.4	(26.9–28.0)	255	26.7	(26.1–27.2)	243
*P* * for trend*	*.38*			<.*001*		
*Marital status*						
Married or cohabiting	27.5	(27.2–27.8)	789	27.8	(27.4–28.1)	722
Single	27.6	(26.8–28.3)	138	27.6	(27.1–28.2)	352
*P* * for trend*	*1.00*			*.70*		

**Table 3:** Odds ratio (OR) and 95% confidence intervals (95% CI) for obesity according to socioeconomic indicators among men.

	Individual effects*	Model 1 (I+II)*	Model 2 (I–III)*	Model 3 (I–IV)*
*I Socioeconomic status in childhood*				
lower	**2.0 (1.2**–**3.3)**	**1.9 (1.1**–**3.3)**	**1.7 (1.0**–**3.1)**	**1.8 (1.0**–**3.1) **
middle	**1.7 (1.0**–**3.0)**	1.7 (0.9–3.0)	1.7 (0.9–3.0)	1.7 (0.9–3.2)
higher	1	1	1	1
*II Education*				
<9 years	**1.6 (1.1**–**2.4)**	**1.4 (1.0**–**2.1)**	1.2 (0.7–1.9)	1.3 (0.7–2.0)
10–12 years	0.9 (0.6–1.3)	0.8 (0.5–1.2)	0.7 (0.5–1.1)	0.8 (0.5–1.2)
≥13 years	1	1	1	1
*III Socioeconomic status in adulthood*				
lower	**1.9 (1.3**–**2.9)**	—	1.6 (0.9–2.7)	1.6 (0.9–2.8)
middle	1.3 (0.9–1.9)	—	1.2 (0.8–1.8)	1.3 (0.8–1.9)
higher	1	—	1	1
*IV Household income*				
Lowest quartile (1st)	1.3 (0.8–2.0)	—	—	0.9 (0.5–1.5)
2nd quartile	1.1 (0.7–1.7)	—	—	0.8 (0.5–1.3)
3rd quartile	0.8 (0.5–1.2)	—	—	0.6 (0.4–1.1)
highest quartile (4th)	1	—	—	1

*Adjusted for age significance was defined as *P* < .05; bolded figures indicate significant associations.

**Table 4:** Odds ratio (OR) and 95% confidence intervals (95% CI) for obesity according to socioeconomic indicators among women.

	Individual effects*	Model 1 (I+II)*	Model 2 (I–III)*	Model 3 (I–IV)*
*I Socioeconomic status in childhood*				
lower	**1.6 (1.0–2.6) **	1.5 (0.9–2.5)	1.2 (0.7–2.0)	1.2 (0.7–2.0)
middle	**1.7 (1.0–2.8)**	1.3 (0.8–2.2)	1.2 (0.7–2.2)	1.2 (0.7–2.2)
higher	1	1	1	1
*II Education*				
<9	**1.7 (1.2**–**2.4)**	**1.7 (1.2**–**2.4)**	**1.5 (1.0**–**2.1)**	1.4 (0.9–2.1)
10–12 years	**1.6 (1.2**–**2.3)**	**1.6 (1.1**–**2.3)**	1.3 (0.9–1.9)	1.3 (0.9–1.9)
≥13 years	1	1	1	1
*III Socioeconomic status in adulthood*				
lower	**2.0 (1.3**–**3.5)**	—	**1.7 (1.0**–**3.0)**	1.5 (0.8–2.6)
middle	**1.6 (1.1**–**2.2)**	—	1.3 (0.9–2.0)	1.3 (0.8–1.9)
higher	1	—	1	1
*IV Household income*				
lowest quartile (1st)	**1.8 (1.2**–**2.6)**	—	—	**1.5 (1.0**–**2.4)**
2nd quartile	**2.0 (1.3**–**3.0)**	—	—	**1.7 (1.1**–**2.6)**
3rd quartile	1.3 (0.8–1.9)	—	—	1.1 (0.7–1.7)
highest quartile (4th)	1	—	—	1

*Adjusted for age significance was defined as *P* < .05; bolded figures indicate significant associations.
